# Supplementary material for: The association of types, intensities and frequencies of physical activity with primary infertility among females in Gaza Strip, Palestine: A case-control study
Source: PLoS One. 2020 Oct 23;15(10):e0241043. doi: 10.1371/journal.pone.0241043 (PMC7584224; doi:10.1371/journal.pone.0241043)
Supplement: S1 File — (DOCX) [file pone.0241043.s001.docx]

Name: ………………………………………… Serial Number: ………………………………………….

Date.: …………………………………………. Contact information: …………………………………

| - **Case Control** | | | |
| --- | --- | --- | --- |
| **Demographic variables:** | | | |
| 1. Age: ………   (Please, record this information from official document if possible) | | | |
| 1. Marital age: ………. | | | |
| 1. Duration of marriage (in years): ……… | | | |
| 1. Age of onset of 1^st^ menstrual period: ………. years old | | | |
| 1. Refugee status: | - Registered refugee - Non-registered refugee - Non-refugee | | |
| 1. Residency: | - North Gaza - Gaza - Middle area - Khan Younis - Rafah | 1. Name of city or village:   …………………………………  ………………………………… | |
| 1. Residency in relation to camps: € Inside camp € Outside camp | | | |
| 1. Site of residency:   (You can mark multiple answers) | - Coastal - Downtown - Industrial area - Agricultural area - Eastern border - Northern border - Southern border - Beside a factory - Beside a landfill | | |
| 1. What type of dwelling unit are you living in?   Caravan\Barracks: Separate established building, usually comprised of one or more rooms. The main construction material of the ceiling and the external walls is made of zinc, tinplate or reinforced fiber | - Villa - House - Apartment - Independent Room - Tent - Marginal\Caravan\Barracks | | |
| 1. What type of dwelling unit you used to live in before marriage? | - Villa - House - Apartment - Independent Room - Tent - Marginal\Caravan\Barracks | | |
| **Socio-economic variables:** | | | |
| 1. Years of schooling completed: | *You:* | | |
|  | - Illiterate □ Secondary - Primary □ Higher education - Preparatory | | |
| 1. Employment status and working field:   (Please, if the answer is Housewife or unemployed, move to Q15) | *You:* | *Your husband:* | |
|  | - Housewife - Employed in public sector - Employed in private sector - Employed in NGOs - Freelancer - Self-employed | - Unemployed - Employed in public sector - Employed in private sector - Employed in NGOs - Freelancer - Self-employed | |
| 1. In which field do you work? | *You:* | | *Your husband:* |
|  | - Computer and Technology - Teaching - Social services - Agriculture - Engineering - Hairdresser - Pharmacist - Physiotherapist - Body fitness trainer - Others (Please, specify): ………………. | | - Computer and Technology - Teaching - Social services - Agriculture - Engineering - Hairdresser - Pharmacist - Physiotherapist - Body fitness trainer - Others (Please, specify): ………………. |
| 1. In which shift do you work? | *You:* | | *Your husband:* |
|  | - Morning shifts - Evening shifts - Night shifts - Rotation Shifts - Split shifts - on-call shifts | | - Morning shifts - Evening shifts - Night shifts - Rotation Shifts - Split shifts - on-call shifts |
| 1. Average amount of household monthly income from all sources: ………… (In NIS please) | | | |
| 1. Do you think it is enough regarding your daily expenses?  - Yes € No | | | |
| 1. Average monthly expenditure on food items: …………. (In NIS please) | | | |
| 1. Average monthly expenditure on non-food items: ………... (In NIS please) | | | |
| 1. What is the major group of expenditure do you exercise as a household?   (Only one answer please) | - Food and Soft Drinks - Tobacco - Clothing and outfits - Housing - Furniture - Medical care - Transportation - Mobiles bills - Education expenses - Loans/Depts | | |

| **Lifestyle:** | |
| --- | --- |
| **Diet:** | |
| 1. **Weight:**   (In Kg, please) | You: …………… |
|  | Your Husband: …………… |
| 1. **Height:**   (In centimeter, please) | You: …………… |
|  | Your Husband: …………… |
| 1. Did you suffer from obesity when you were child? | You: € Yes € No |
|  | Your Husband: € Yes € No |
| 1. Are you allergic to any food item/s?   (If no, please go to Q98) | You: € Yes € No |
|  | Your Husband: € Yes € No |
| 1. If yes, please specify: | You: ………………………………………... |
|  | Your Husband: ………………………………. |

**Short Physical activity questionnaire:**

I am going to ask you about the time you spend doing different types of physical activity in a typical week.

***The first question:*** is about the time you spent sitting during the last 7 days include time spent at work, at home, while doing course, work, and during leisure time. This may include time spent sitting at a desk, visiting friend, reading or setting or lying down to watch television (Sedentarily)

| During the last 7 days, how much time did you spend sitting during a day? | Hours: …………. | Minute: ……… | - Don’t know | P1 |
| --- | --- | --- | --- | --- |

***The second question:*** is about the time you spent walking in the last 7 days this include at work and at home, walking to travel from place to place and any other walking that you might do solely for recreation sport, exercise or leisure

| During the last 7 days on how many days did you walk for at least 10 minutes at a time? | Days: …………. | - No days | - Don’t know | P2 |
| --- | --- | --- | --- | --- |
| How much time did you usually spend walking on one of those days? | Hours: …………. | Minute: ……… | - Don’t know | P3 |

***The third question:*** during the last days, on how many days did you do moderate physical activity like gardening, cleaning, bicycling at regular pace, swimming or other fitness activities (do not include walking).

| **Think only about those**  Physical activities that you did for at least 10 minutes. | Days: …………. | - No days | - Don’t know | P4 |
| --- | --- | --- | --- | --- |
| How much time did you usually spend doing moderate activities on one of those days? | Hours: …………. | Minute: ……… | - Don’t know | P5 |

***The fourth question:*** during the last 7 days on how many days did you do vigorous physical activity like heavy lifting heavier garden or construction work, aerobic jogging, running or fast bicycling

| **Think only about those**  Physical activities that you did for at least 10 minutes at a time. | Days: …………. | - No days | - Don’t know | P6 |
| --- | --- | --- | --- | --- |
| How much time did you usually spend doing vigorous physical activities on one of those days? | Hours: …………. | Minute: ……… | - Don’t know | P7 |

الاسم: ............................. الرقم التسلسلي: ...................................

التاريخ: ........................... معلومات للتواصل:................................

| - **Case € Control** | | |
| --- | --- | --- |
| **متغيرات ديموغرافية:** | | |
| 1. العمر: ..........   (الرجاء تسجيل هذه المعلومات من وثائق رسمية) | | |
| 1. العمر عند الزواج: ............... | | |
| 1. مدة الزواج ( بالسنوات ): ................ | | |
| 1. سن بداية الدورة الشهرية الأولى: .......... بالسنوات | | |
| 1. حالة اللجوء: | - لاجئ مسجل - لاجئ غير مسجل - غير لاجئ | |
| 1. مكان السكن: | - شمال غزة - غزة - المنطقة الوسطى - خانيونس - رفح | 1. اسم المدينة او القرية :   .............................  ............................. |
| 1. مكان السكن بالنسبة للمخيمات: € داخل المخيمات € خارج المخيات | | |
| 1. موقع السكن:   (إحتمال أكثر من إجابة واحدة) | - الساحل - وسط المدينة - منطقة الصناعية - منطقة زراعية - الحدود الشرقية - الحدود الشمالية - الحدود الجنوبية - بجوار مصنع - بجوار مكب نفايات | |
| 1. ما نوع الوحدة السكنية الذي تعيش فيه حاليا؟   براكيه/ كرفان/ بركس: هو مبنى قائم بذاته مؤقت وغير تقليدي للسكن، ويتكون من غرفة واحدة أو اكثر وتكون المادة الغالبة للجدران الخارجية والسطح من الزنك (الصاج) أو التنك أو الاسبست**،** أو البلاستيك المقوى (فيبر كلاس) أو الخشب | - فيلا - منزل - شقة - غرفة مستقلة - خيمة - الهوامش/ منزل متنقل/ باراكس | |
| 1. ما نوع الوحدة السكنية قبل الزواج ؟ | - فيلا - منزل - شقة - غرفة مستقلة - خيمة - براكيه/ كرفان/ بركس | |
| **متغيرات إجتماعية و إقتصادية:** | | |
| 1. درجة التعليم : | *الزوجة:* | |
|  | - غير متعلم - ابتدائي - إعدادي - ثانوي - جامعة   تعليم عالي | |
| 1. الوظيفة أو مجال العمل: | *الزوجة:* | *الزوج:* |
|  | - ربة منزل - موظفة في القطاع العام - موظفة في القطاع الخاص - موظفة في المؤسسات الغير حكومية - عمل بالقطعة - العمل للحساب الخاص | - عاطل عن العمل - موظف في القطاع العام - موظف في القطاع الخاص - موظف في المؤسسات الغير حكومية - عمل بالقطعة - العمل للحساب الخاص |
| 1. ما طبيعة عملك؟ | *الزوجة:* | *الزوج:* |
|  | - الكمبيوتر و تكنولوجيا المعلومات - التعليم - الخدمات الاجتماعية - الزراعة - الهندسة - مصفف شعر - الصيدلة - العلاج الطبيعي - مدرب لياقة بدنية - أخرى (الرجاء حدد): ...................... | - الكمبيوتر و تكنولوجيا المعلومات - التعليم - الخدمات الاجتماعية - الزراعة - الهندسة - مصفف شعر - الصيدلة - العلاج الطبيعي - مدرب لياقة بدنية - أخرى (الرجاء حدد): ...................... |
| 1. توقيت العمل؟ | *الزوجة:* | *الزوج:* |
|  | - فترة صباحية - فترة مسائية - قترة ليلية - نظام مناوبات - ساعات مجزءة - على الطلب | - فترة صباحية - فترة مسائية - قترة ليلية - نظام مناوبات - ساعات مجزءة - على الطلب |
| 1. متوسط مقدار دخل الأسرة الشهري من جميع المصادر: .................. (بالشيقل رجاءً) | | |
| 1. هل تظنين أن الدخل الشهري كاف بالنسبة للنفقات اليومية؟  - نعم € لا | | |
| 1. متوسط الإنفاق الشهري على المواد الغذائية: ................ (بالشيقل رجاءً) | | |
| 1. متوسط الإنفاق الشهري على المواد غير الغذائية: ................ (بالشيقل رجاءً) | | |
| 1. ما هي المجموعة الرئيسية من النفقات التي تمارسونها كأسرة؟   (إجابة واحدة فقط من فضلك) | - الطعام والمشروبات - الدخان - الملابس - اعمار السكن - الأثاث - الرعاية الصحية - التنقل والمواصلات - فواتير الهواتف - مصاريف التعليم - القروض | |

| **نمط المعيشة:** | |
| --- | --- |
| **الغذاء** | |
| 1. **الوزن رجاءً:**   (بالكيلوغرام) | الزوجة :.................................................... |
|  | الزوج : ..................................................... |
| 1. **الطول رجاءً:**   (بااسنتيمتر) | الزوجة :.................................................... |
|  | الزوج : ..................................................... |
| 1. هل عانيت من السمنة بعمر الطفولة ؟ | الزوجة: € نعم € لا |
|  | الزوج : € نعم € لا |
| 1. هل لديك حساسية لنوع محدد من الأطعمة؟ | الزوجة: € نعم € لا |
|  | الزوج : € نعم € لا |
| 1. إذا يوجد حساسية, الرجاء تحديد المسبب: | الزوجة :.................................................... |
|  | الزوج : ..................................................... |

إستبيان النشاط البدني القصير:

سوف أقوم بالسؤال عن الوقت الذي تقضيه في القيام بأنواع مختلفة من النشاط البدني في أسبوع نموذجي.

***السؤال الأول:*** هو الوقت الذي قضيته في الجلوس خلال آخر 7 أيام ، ويشمل الوقت الذي قضيته في العمل ، في المنزل ، أثناء القيام بدورة ، وأثناء وقت الفراغ. قد يشمل ذلك الوقت الذي تقضيه في الجلوس على مكتب ، زيارة صديق ، القراءة أو الجلوس أو الاستلقاء لمشاهدة التلفزيون.

| خلال آخر 7 أيام ، كم من الوقت قضيته جالسًا خلال اليوم؟ | ساعات: .......... | دقائق: ............. | لا أعلم | P1 |
| --- | --- | --- | --- | --- |

***السؤال الثاني:*** هو الوقت الذي قضيته في المشي في آخر 7 أيام ، ويشمل ذلك في العمل والمنزل ، والمشي للتوجه من مكان إلى مكان وأي نوع اخر من المشي الذي قد تفعله فقط لممارسة الرياضة أو الترفيه.

| خلال آخر 7 أيام كم عدد الأيام التي سرت فيها لمدة 10 دقائق على الأقل في المرة الواحدة؟ | أيام: ........... | ولا يوم | لا أعلم | P2 |
| --- | --- | --- | --- | --- |
| كم من الوقت قضيت عادة في المشي في أحد تلك الأيام؟ | *ساعات: ..........* | *دقائق: .............* | *لا أعلم* | *P3* |

***السؤال الثالث:*** خلال الأيام الأخيرة ، كم عدد الأيام التي مارست فيها نشاطًا بدنيًا معتدلًا مثل البستنة أو التنظيف أو ركوب الدراجات الهوائية بوتيرة منتظمة أو السباحة أو غيرها من أنشطة اللياقة البدنية (لا تشمل المشي).

| فكر فقط في الاتي.  الأنشطة البدنية التي قمت بها لمدة 10 دقائق على الأقل. | أيام: ........... | ولا يوم | لا أعلم | P4 |
| --- | --- | --- | --- | --- |
| كم من الوقت تقضيه عادة في القيام بأنشطة معتدلة في أحد تلك الأيام؟ | *ساعات: ..........* | *دقائق: .............* | *لا أعلم* | *P5* |

***السؤال الرابع:*** خلال آخر 7 أيام ، كم عدد الأيام التي مارست فيها نشاطًا بدنيًا قويًا ، مثل: أعمال البناء الثقيلة أو رفع الأحمال الثقيلة أو الركض أو ركوب الدراجات الهوائية السريعة

| فكر فقط في الاتي.  الأنشطة البدنية التي قمت بها لمدة 10 دقائق على الأقل. | أيام: ........... | ولا يوم | لا أعلم | P6 |
| --- | --- | --- | --- | --- |
| ما مقدار الوقت الذي تقضيه عادة في ممارسة الأنشطة البدنية القوية في أحد تلك الأيام؟ | *ساعات: ..........* | *دقائق: .............* | *لا أعلم* | *P7* |
