# Supplementary material for: The association of types, intensities and frequencies of physical activity with primary infertility among females in Gaza Strip, Palestine: A case-control study
Source: PLoS One. 2020 Oct 23;15(10):e0241043. doi: 10.1371/journal.pone.0241043 (PMC7584224; doi:10.1371/journal.pone.0241043)
Supplement: S2 File — (DOCX) [file pone.0241043.s002.docx]

**Readme Document**

*Data from the study of physical activity among infertile and fertile females*

*International Physical Activity Questionnaire (IPAQ) - WHO*

**Categorical scoring of physical activity:**

1. **Category 1 High**

At least one of two criteria should be fulfilled:

1. vigorous-intensity activity on at least 3 days achieving a minimum Total
   physical activity of at least 1500 MET-minutes/week.
2. 7 or more days of any combination of walking, moderate-intensity or
   vigorous-intensity activities achieving a minimum Total physical activity
   of at least 3000 MET-minutes/week.
3. **Category 2 Moderate**

At least one of three criteria should be fulfilled:

1. 3 or more days of vigorous-intensity activity of at least 20 minutes per day.
2. 5 or more days of moderate-intensity activity and/or walking of at least 30
   minutes per day.
3. 5 or more days of any combination of walking, moderate-intensity or vigorous
   intensity activities achieving a minimum Total physical activity of at least 600
   MET-minutes/week.
4. **Category 3 Low**

Those individuals who not meet criteria for Categories 2 or 3 are considered to have a ‘low’ physical activity level.

**Continuous scoring of physical activity:**

1. Walking MET-minutes/week = 3.3 * walking minutes * walking days
2. Moderate MET-minutes/week = 4.0 * moderate-intensity activity minutes * moderate days
3. Vigorous MET-minutes/week = 8.0 * vigorous-intensity activity minutes * vigorous-intensity days
4. Total physical activity MET-minutes/week = sum of Walking + Moderate + Vigorous MET minutes/week scores.

| Variable | Stands for | Description/Equation |
| --- | --- | --- |
| SN | Serial Number |  |
| CC | Case/Control | 1 = Case  2 = Control |
| ccc | Control/Case | 1 = Control  2 = Case |
| Age | Female age | Continuous |
| Age.G | Female age - Grouping 1 | 1 = (< 30); 2 = (≥ 30) |
| Age.G2 | Female age - Grouping 2 | 1 = (< 25); 2 = (≥ 25) |
| M.age | Marital Age | Continuous |
| M.age.G | Marital age - Grouping | 1 = (< 18); 2 = (18-28); 3 = (≥ 29) |
| MnAg | First menses age | Continuous |
| MnAg.G | First menses age - Grouping | 1 = (<14); 2 = (≥ 14) |
| Ref | Refugee status | 1= (Refugee); 2= (Non-Refugee) |
| R.Cmp | Residency-camp | 1= (Living inside camps); 2= (Living outside camps) |
| R.Tn | Residency-Downtown | 1= (Living downtown); 2= (living elsewhere) |
| Ed | Years of schooling | 1= Illiterate  2= Primary  3= Secondary  4= High school  5= University  6= Higher education |
| Ed.G | Years of schooling - Grouping | 1= ≤ preparatory; 2= High school; 3≥ University |
| Emp | Employment | 1=Housewife  2=Employed in public sector  3=Employed in private sector  4=Employed in NGOs  5=Freelancer  6=Self-employed |
| Emp.G | Employment - Grouping | 1=Employed  2=Unemployed |
| EmpH | Husband Employment | 1=Unemployed  2=Employed in public sector  3=Employed in private sector  4=Employed in NGOs  5=Freelancer  6=Self-employed |
| EmpH.G | Husband Employment - Grouping | 1=Employed  2=Unemployed |
| Inc | Monthly income | Continuous |
| Inc.G | Monthly income – Grouping  In NIS (New Israeli Shekel) | 1= (≤1500 NIS)  2= (1500-2500 NIS)  3= (>2500 NIS) |
| P1 | Sitting per day in minutes | Time spent sedentarily per day in minutes |
| P2 | Walking days/week | Number of days walking per week |
| P3 | Walking per day in minutes | Time spent walking per day in minutes |
| P4 | Moderate-days/week | Number of days per week doing moderate activities |
| P5 | Moderate/d Min | Time spent per day in moderate activities in min |
| P6 | Vigorous-days/w | Number of days per week doing vigorous activities |
| P7 | Vigorous/d Min | Time spent per day in vigorous activities in min |
| WMET | Walking MET-min/w | WMET = 3.3 * P3 * P2 |
| MMET | Moderate MET- min/w | MMET = 4 * P5 * P4 |
| VMET | Vigorous MET – min/w | VMET = 8 * P7 * P6 |
| TMET | Total physical activity MET – min/w | TMET = WMET + MMET + VMET |
| Atleast7 | At least 7 days of any combination of walking, moderate-intensity or vigorous-intensity activities | Atleast7 = [(P2 + P4 + P6) ≥ 7]  1 = At least 7 days  0 = Less than 7 days |
| H1 | High category – Criterion “a” | H1 = (P6 ≥ 3) & (TMET ≥ 1500)  1 = Meet criteria  0 = Do not meet criteria |
| H2 | High category – Criterion “b” | H2 = (Atleast7 = 1) & (TMET ≥ 3000)  1 = Meet criteria  0 = Do not meet criteria |
| High | High physical activity | High = (H1 = 1) or (H2 = 1)  1 = Meet criteria  0 = Do not meet criteria |
| Atleast5 | At least 5 days of any combination of walking, moderate-intensity or vigorous-intensity activities | Atleast5 = [(P2 + P4 + P6) ≥ 5]  1 = At least 5 days  0 = Less than 5 days |
| M1 | Moderate category – criterion “a” | M1 = (P6 ≥ 3) & (P7 ≥ 20)  1 = Meet criteria  0 = Do not meet criteria |
| M2 | Moderate category – criterion “b” | M2 = (Atleast5 = 1) & (P3+P5 ≥ 30)  1 = Meet criteria  0 = Do not meet criteria |
| M3 | Moderate category – criterion “c” | M3=(Atleast5=1)&(TMET >= 600)  1 = Meet criteria  0 = Do not meet criteria |
| Mod | Moderate physical activity | IF(High=0), Mod=(M1=1)or(M2=1)or(M3=1)  1 = Meet criteria  0 = Do not meet criteria |
| Low | Low physical activity | Low = 1 If [(High + Mod) = 0]  1 = Low physical activity |
| Cat.PA | All categories in one column | 1 = High physical activity  2 = Moderate physical activity  3 = Low physical activity |
| Wt | Weight of females in Kg | Continuous |
| Kcal.W | Kilocalories exerted in Walking MET-minutes/week | Kcal.W = WMET x (Weight / 60 kg) |
| Kcal.M | Kilocalories exerted in Moderate MET-minutes/week | Kcal.M = MMET x (Weight / 60 kg) |
| Kcal.V | Kilocalories exerted in Vigorous MET-minutes/week | Kcal.V = VMET x (Weight / 60 kg) |
| T.Kcal | Total kilocalories exerted / week | T.Kcal = Kcal.W + Kcal.M + Kcal.V |
| T.Kcal.G | Total kilocalories exerted/w - Grouping | 1 = < 3000 / week  2 = ≥ 3000 / week |
| P1.G | Sitting per day in minutes - Grouping | 1 = > 300 min/day  2 = ≤ 300 min/day |
