# Supplementary material for: The association of types, intensities and frequencies of physical activity with primary infertility among females in Gaza Strip, Palestine: A case-control study
Source: PLoS One. 2020 Oct 23;15(10):e0241043. doi: 10.1371/journal.pone.0241043 (PMC7584224; doi:10.1371/journal.pone.0241043)
Supplement: Attachment — Submitted filename: Response to Reviewers.docx [file pone.0241043.s005.docx]

Dear academic editor and reviewers,

Please, let us thank you for your time and effort in editing and reviewing our manuscript and please, kindly find replies for your comments:

- Please include additional information regarding the survey or questionnaire used in the study and ensure that you have provided sufficient details that others could replicate the analyses. For instance, if you developed a questionnaire as part of this study and it is not under a copyright more restrictive than CC-BY, please include a copy, in both the original language and English, as Supporting Information.

Questionnaire is added as a supporting information. Also, the study dataset was previously uploaded in the supporting information.

- Please state how many participants were included in the pilot testing of this tool.

Added in line 195

- Please provide further details of the experts employed to develop this questionnaire. For example, their levels of expertise, ages and whether or not they provided written, informed consent.

The questionnaire was self-developed except for the part that includes physical examination part in which it was adopted from the Short Form of International Physical Activity Questionnaire of the World Health Organization template. The whole tool is uploaded as a supporting information.

3. Please include captions for your Supporting Information files at the end of your manuscript, and update any in-text citations to match accordingly.

Reviewer #1:

1) What is this study's hypothesis? Authors need to write this in the Introduction.

The study hypothesis has been added to the introduction (lines 122 - 126). Thanks for your comments.

2) The information of the participant’s selection is not clear. So, please add this information as a Flow chart in the results.

A flow chart has been added to the methodology section at the study participants and sampling part as Fig 1. Thank you for your enriching comments.

Reviewer #2: Manuscript Number: PONE-D-20-17392


Main comments:

- The authors did not report any information about BMI or anthropometric status. These data are essential when dealing with physical activity or sedentary

Yes, you are right. It is crucial to explore anthropometric measures when we are dealing with physical activity. We have added information related to weight, BMI and childhood obesity to the results and discussion section (lines 269 - 281). Thanks for the comment.

- The authors do not provide information on previous pregnancies for the infertile women and the ages of children of fertile women. Have they had miscarriages? Previous children?

The cases were selected upon a criterion of having primary infertility. To be primary infertile they will have a zero history of pregnancies. For fertile women, the selection criteria included those who had no previous or current bad obstetric and perinatal histories. That’s why we did not inquire about previous miscarriage.

- The conclusion is too peremptory: the study shows an association between women's physical activity and fertility, but not a causal link. This would require a prospective interventional study. The authors could modulate their conclusion accordingly.

Yes, actually it is correct and we should not have been so definitive in stating the conclusion. Observational studies are suggestive. We have modulated the conclusion accordingly (Lines 388 – 393).

Minor comments:

- The authors report no information about male partner

Actually, we did not include the male partner in this study.

- Line 132: why did the inclusion criteria for fertile women require two pregnancies? One pregnancy is enough to “label fertility”. Would it have been more appropriate to use the time to pregnancy and the date of the last pregnancy?

We have chosen controls upon which those who had at least two successful pregnancies without assistive reproductive techniques to exclude secondary infertility. Other inclusion criteria for controls was to have no bad obstetric or perinatal history like miscarriage, still births, early perinatal deaths but this was not mentioned in the methodology. It has been added, so that the selection criteria would be clearer. Regarding time to pregnancy, we considered the available cultural norms and traditions unique to this community. Here, couples do not delay planning to pregnancy once they are married and they are always anxious and otherwise would seek medical advice immediately. Still this could be mentioned as a limitation. Thanks for your enriching comments.

Finally, I would like to thank you all for the effort and time and for enriching our research with your valuable comments.

Best regards,
